# Low fat diets increase survival of a mouse model of spinal muscular atrophy

**DOI:** 10.1002/acn3.50920

**Published:** 2019-10-13

**Authors:** Marc‐Olivier Deguise, Lucia Chehade, Alexandra Tierney, Ariane Beauvais, Rashmi Kothary

**Affiliations:** ^1^ Regenerative Medicine Program Ottawa Hospital Research Institute Ottawa Ontario Canada K1H 8L6; ^2^ Department of Cellular and Molecular Medicine University of Ottawa Ottawa Ontario Canada K1H 8M5; ^3^ Centre for Neuromuscular Disease University of Ottawa Ottawa Ontario Canada K1H 8M5; ^4^ Department of Biochemistry, Microbiology, and Immunology Faculty of Medicine, and Department of Medicine University of Ottawa Ottawa Ontario Canada K1H 8M5

## Abstract

Spinal muscular atrophy (SMA) is a neuromuscular disorder leading to paralysis and death. Recent evidence shows increased susceptibility to dyslipidemia and liver steatosis in patients. Here, we provide evidence that low fat diets nearly double survival in *Smn^2B/−^* mice, a model for SMA, independent of changes in SMN levels, liver steatosis, or enhanced hepatic functions. Liver damage and ketone levels were reduced, implying a lower reliance on fatty acid oxidation. This preclinical proof of concept study provides grounds for controlled clinical investigation of dietary needs and offers evidence to inform nutritional guidelines specific to SMA.

## Introduction

Spinal muscular atrophy (SMA) is a devastating neurological disease affecting patients of all ages.^1^ Commonly affecting young infants, SMA results in motor neuron loss leading to generalized weakness and paralysis.^1^ The genetic basis of SMA is deletion or mutation in the ubiquitously expressed *Survival motor neuron 1* (*SMN1*) gene,^2^ which encodes a protein (SMN) involved in housekeeping functions, including RNA metabolism and splicing.^3^

Over time, defects in multiple non‐neuronal cell types have been identified in SMA.^4^, ^5^ Metabolic defects were suspected even prior to the identification of the SMA disease gene.^6^ In early clinical studies, the presence of dicarboxylic aciduria, reduced beta‐oxidation capacity, and a case report of fatty liver highlighted the potential deficits in fatty acid metabolism.^6^, ^7^, ^8^ Recently, we identified the increased susceptibility of SMA patients and preclinical models to develop dyslipidemia and fatty liver.^9^ More specifically, 13% of a SMA patient cohort showed alterations in more than three common measures of dyslipidemia (total cholesterol, low‐density lipoprotein (LDL), high‐density lipoprotein (HDL), triglycerides (TGs), and non‐HDL cholesterol), which is a greater prevalence than in the normal population.^10^, ^11^ Moreover, about one‐third of patients' autopsies showed liver steatosis (commonly known as fatty liver), a feature that less than 0.7% of 2‐ to 4‐year‐old normal children harbor.^12^ In accordance with these clinical findings, all *Smn* ^2^ *^B/−^* mice, a commonly used model of SMA that lives about 25 days of age, developed a nonalcoholic fatty liver phenotype and dyslipidemia.^9^ These findings raise concerns for additional comorbidities such as metabolic syndrome, cardiovascular disease, and stroke in a subset of SMA patients as they age.

Here, we show that low fat diets were sufficient to double lifespan in *Smn^2B/−^* mice, without any reduction in the increased hepatic TG content, which suggests that there was no significant change in the fatty liver pathology. Liver function, such as protein output, also showed no amelioration. Some improvements were identified in liver damage markers, and in glucagon‐like peptide 1 (GLP‐1) and ketone levels, which trended toward normal in low fat and high‐sucrose diet (HSD). Altogether, this work highlights the need for additional controlled nutritional preclinical and clinical research. Nutritional guidelines specific to SMA should then be further developed from these evidence‐based studies.

## Materials and Methods

### Mouse models

The *Smn^2B/−^* (C57BL/6J background)^13^ mice were housed at the University of Ottawa and cared for according to the Canadian Council on Animal Care. Tissues were collected ad libitum between 9 and 11 am to limit the effect of the circadian rhythm.

### Diet modulation

Dams were provided excess normal chow (NC) (Teklad Global 18% Protein Rodent Diet), high‐fat diet (HFD) (Research Diets D12492), low‐fat diet (LFD) (Research Diets D12450J), or high‐sucrose/low‐fat diet (HSD) (Research Diets D12450B) at the bottom of the cage 2 weeks after putting the breeding pair together, which was changed every 2 days. Following weaning, mice were provided with the diets until endpoint. At least three males and three females were included in each group to account for sex differences.

### Gene expression studies

RNA extraction, cDNA reverse transcription, and qPCR were performed as previously published.^9^ Primers are as follows: Ywhaz forward 5’ AAG ACA GCA CGC TAA TAA TGC 3’, reverse 5’ TTG GAA GGC CGG TTA ATT TTC 3’, hprt1 forward 5’ CCC AGC GTC GTG ATT AGT GAT G 3’, reverse 5’ TTC AGT CCT GTC CAT AAT CAG TC 3’, and plin2 forward and reverse primer were ordered from primePCR (Biorad ‐ qMmuCIP0033479). Results were normalized with two genes identified as appropriate stable internal reference given M value below 0.5 and coefficient of variance below 0.25.

### Immunoblotting

Western blots were performed as previously described.^9^ Results were normalized to total protein.

### Lipid quantification and blood chemistry

Tissue lipid analysis was performed at Vanderbilt Mouse Metabolic Phenotyping Center.^9^ All blood collected in this study was sampled randomly (i.e., no fasting period) between 9 and 11 am. Alanine aminotransferase (ALT), total protein, and non‐esterified fatty acids (NEFA) were assessed at Comparative Clinical Pathology Services, LLC., Columbia, Missouri. Glucagon and GLP‐1 quantification were outsourced to Eve Technologies Corp. (Calgary, Alberta).^9^ Ketone and glucose were analyzed with Freestyle Precision Neo Blood Glucose and Ketone monitoring system.

### Statistics

Data are presented as the mean ± standard error of the mean. One‐way ANOVA analysis was used with Tukey as posttest. Survival curves were compared using a Mantel‐Cox test. Statistical analysis was performed in GraphPad Prism. Grubbs test (alpha = 0.05) was performed to identify outliers, which were removed from the analysis. Significance was set at *P* ≤ 0.05 for *, *P* ≤ 0.01 for **, *P* ≤ 0.001 for ***, and *P* ≤ 0.0001 for ****. *N* number for each experiment is as indicated in the figure legends.

### Data and materials availability

Raw data can be provided upon request.

## Results

### low‐fat diet leads to increased survival without a correction of hepatic triglyceride or liver function

**Figure 1:**
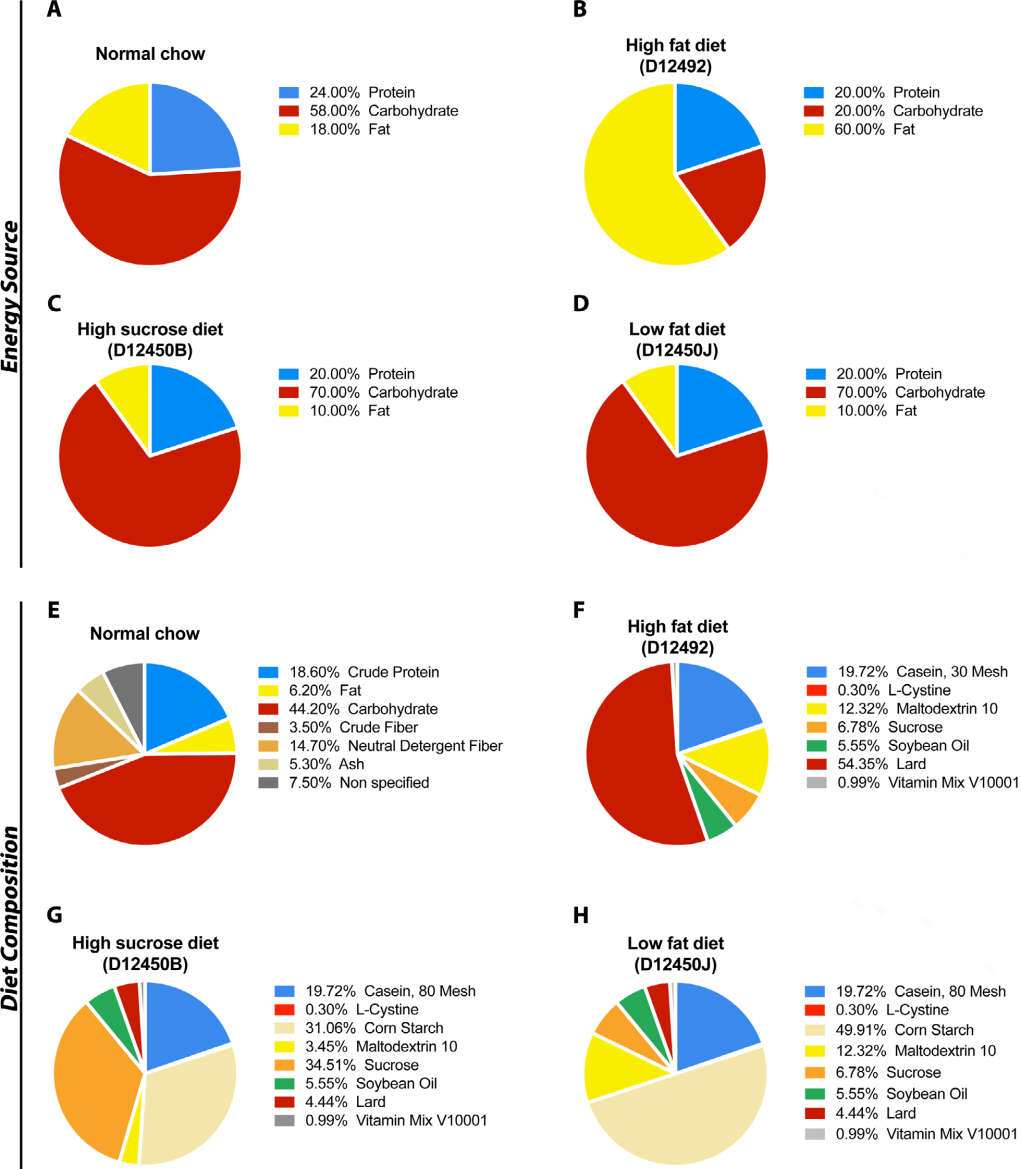
Composition of diets used in this study. Energy sources for normal chow (A), high‐fat diet – Research Diets D12492 (B), high‐sucrose diet – Research Diets D12450B (C), and low‐fat diet – Research Diets D12450J (D) are presented. The diet content is shown for normal chow (E), high‐fat diet – Research Diets D12492 (F), high‐sucrose diet – Research Diets D12450B (G), and low‐fat diet – Research Diets D12450J (H).

**Figure 2:**
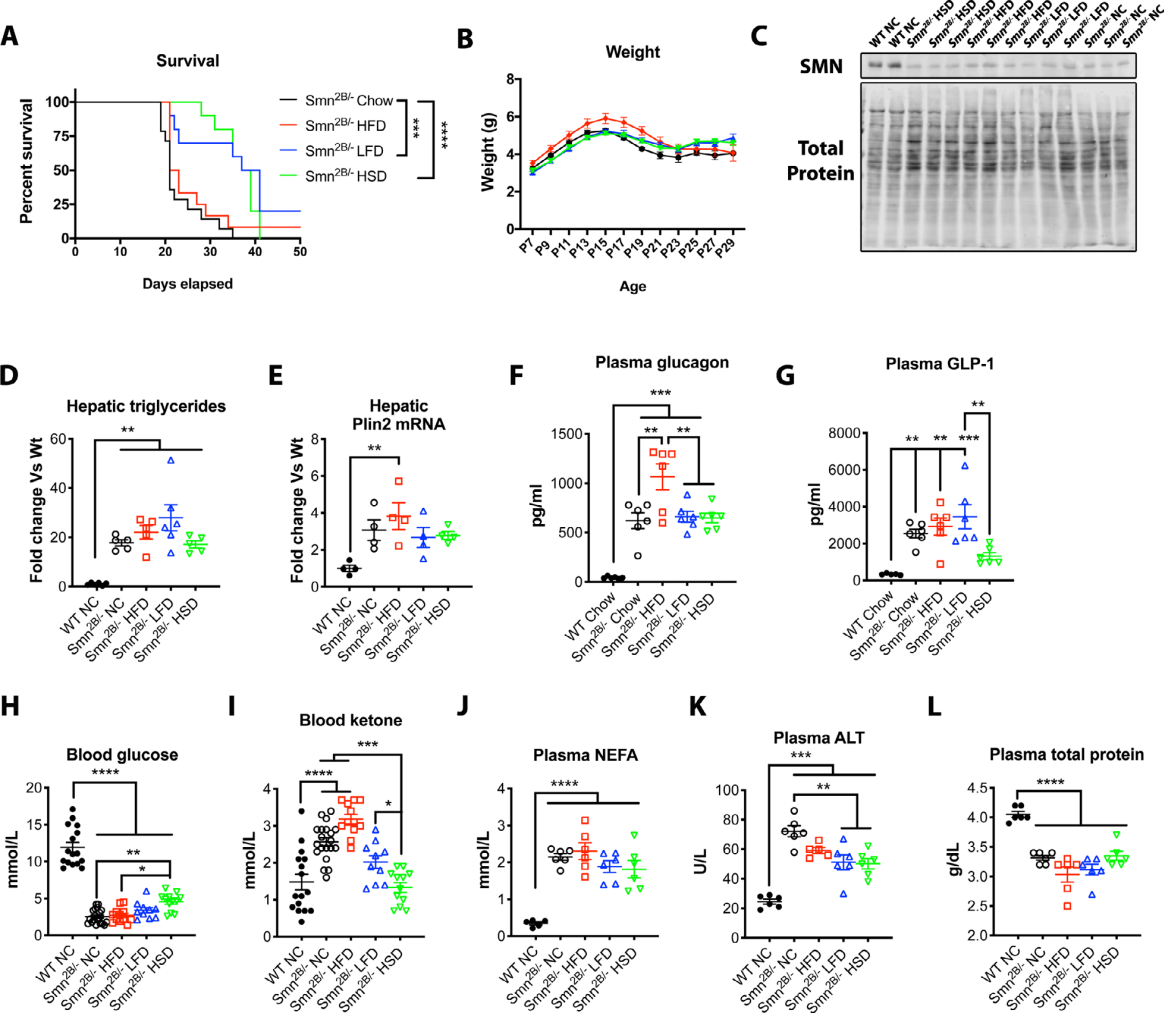
Low fat diets enhance survival by switching cell metabolism away from fat as an energy source. (A) LFD and HSD lead to a median survival of 38 and 39 days for *Smn^2B/−^* mice in comparison to 21 and 22 days when fed NC and HFD, respectively. (B–E) Weight, hepatic SMN, hepatic triglycerides, and plin2 mRNA levels were unchanged by the introduction of different diets in *Smn^2B/−^* mice. (F) Glucagon was further elevated by HFD diet in *Smn^2B/−^* mice while no change was observed by the introduction of other diets. (G) GLP‐1 was considerably diminished upon administration of HSD but not with other diets. (H) Glucose was mostly unchanged by diet modulation, albeit slightly elevated in HSD group. (I) Levels of ketone bodies were enhanced further by introduction of HFD, significantly diminished by LFD diet, while back to normal with the HSD diet in *Smn^2B/−^* mice. (J) Non‐esterified fatty acids were unchanged upon diet modulation, albeit slightly reduced in the HSD cohort. (K) Significant reduction in plasma ALT levels was observed in LFD and HSD cohort, supporting reduced hepatocyte damage. (L) Liver function, as shown by total protein output, was not restored in LFD and HSD cohorts**.** qPCR data were normalized with HPRT1 and Ywhaz in (E) (*N* value for each experiment is as follows: *N* = 10–19 for A‐B, 3‐6 for C–G & J–M, 10–20 for H‐I, one‐way ANOVA with Tukey's correction, Grubbs test (alpha – 0.05) for outliers, *P* ≤ 0.05 for *, *P* ≤ 0.01 for **, *P* ≤ 0.001 for *** and *P* ≤ 0.0001 for ****).

We have previously shown that the *Smn^2B/−^* mice develop fatty liver, with over 25‐fold more TGs in their liver than control mice.^9^ At this time, it is unclear whether this is contributing to disease severity and whether it could be modulated through pharmacological or dietary means. Dietary intake plays an important part in liver lipid accumulation.^14^, ^15^ Additionally, many families find subjective clinical benefit for SMA children in adhering to a low‐fat diet called the “amino acid diet” (http://www.aadietinfo.com). As such, we tested three different diets: HFD, LFD, and HSD, which were compared to standard NC (Fig. 1). Given the short lifespan of *Smn* ^2^ *^B/−^* mice, diets were administered to the dams until the pups could freely feed on their own. It has previously been shown that milk of rat dams fed HFD had higher fat content than those on normal chow by 21 days postpartum, while their offspring showed increased weights, change in body composition, and metabolic abnormalities.^16^ Surprisingly, introduction of LFD and HSD doubled life expectancy of *Smn^2B/−^* mice in comparison to normal chow (median lifespan of 38 and 39 vs. 21 days, respectively) (Fig 2A). Note, both LFD and HSD have similar fat and carbohydrate proportion as energy source but differ significantly in the type of carbohydrate they provide (Fig. 1). HFD (median lifespan of 22 days) did not lead to improvement (Fig. 2A). Interestingly, the weight of the mice, SMN levels, or hepatic fat content (TG levels and plin2 mRNA levels^17^) were only marginally changed regardless of diet (Fig. 2B–E). Hyperglucagonemia was previously observed in the *Smn^2B/−^* mice.^18^ While plasma glucagon levels remain constant (Fig. 2F), a reduction of GLP‐1 in the HSD group, a resultant protein from the proglucagon produced in the intestines, was observed (Fig. 2G). We have previously noticed sustained low glucose^9^ and high circulating NEFA, a product from peripheral lipolysis of white adipose tissue (data not shown). We next speculated that LFD and HSD may simply restore appropriate proportions of energy substrate for usage, by reducing circulating fat and increasing glucose. Indeed, we noted a modest increase in plasma glucose levels but sustained NEFA in *Smn^2B/−^* mice on the HSD regimen (Fig. 2H,J). More importantly, plasma ketones were reduced in both LFD and HSD cohorts, implying reduced beta‐oxidation and a lower reliance on fatty acids as an energy source (Fig. 2I). Interestingly, liver damage was diminished in *Smn^2B/−^* fed LFD and HSD diets in comparison to *Smn^2B/−^* mice on normal chow, as assessed by plasma ALT levels (Fig. 2K). However, low hepatic function remained as total protein production (Fig. 2L) and *Igf1* mRNA production (data not shown) were unchanged amongst the different *Smn^2B/−^* diet cohorts. Altogether, diet modulation can significantly improve the lifespan of *Smn^2B/−^* mice, likely by altering mitochondrial bioenergetic substrate utilization, without influence on hepatic fat accumulation.

## Discussion

Consensus on standards of care in SMA^19^, ^20^ repeatedly highlighted the need for more research in SMA metabolism. Despite this, optimal nutritional guidelines for SMA patients are still lacking. Currently, many SMA patients are either malnourished, underfed, or overfed.^21^, ^22^ Over the years, many SMA families have adopted the “amino acid diet.” This diet was developed purely based on observations and experiences of caregivers, and consists of reduced fat intake consumption (10–20%) and elemental free amino acid formula amongst other components. Nevertheless, scientific and preclinical evidence on the benefit of this diet is lacking.

In our study, it was intriguing that low‐fat/low‐sucrose diet or low‐fat/HSD led to doubling of the lifespan in a mouse model of SMA. Interestingly, it was previously speculated that a high‐carbohydrate/low‐fat diet would provide energy substrates that do not depend on fatty acid oxidation, keep the level of free fatty acids under control, and diminish production of dicarboxylic acid in the circulation – factors thought to have toxic potential to SMA patients.^7^, ^23^ This is consistent with some aspects of the “amino acid” diet. A LFD/high‐carbohydrate diet was tested in 13 SMA patients in the 1990s.^24^ While the authors claimed a beneficial outcome, there was controversy about the classification scheme used, given the lack of genetic diagnostic tools.^7^ Hence, the interpretation of the results is difficult^7^ and the study has not since been reproduced. One previous study concluded that higher fat content may confer protective benefits.^25^ However, the diets used consisted of two different chows with many differences amongst them, did not control exclusively for the fat content, consisted of only a 5% fat difference, and showed marginal difference in survival (3 days).^25^ In our study, the use of “research diets” minimized the effect of other substances while maximizing the differences in fat intake or sucrose, especially fat content. Nevertheless, it is unknown whether the full extent of the nutritional content of each diet was carried in the milk of the dams. On another note, it was interesting to note that hepatic TGs did not change significantly upon diet modulation. However, it is thought that dietary lipids only contribute to 15% of lipid accumulation in the liver in nonalcoholic fatty liver disease.^26^ As such, it appears that diet modulation simply decreases fatty substrate availability coming directly from dietary intake, diminishing the load on fatty acid oxidation, as shown by normalization of ketone bodies. While we were able to identify a clear difference between high‐ and low fat diets (LFD and HSD vs. HFD), there is a more subtle difference apparent between the effects of LFD and HSD. This may be attributed to the different composition of carbohydrate in the respective diets, even though the carbohydrate provided the same equivalence of energy. HSD contains sucrose as its major carbohydrate while the LFD major carbohydrate was cornstarch. This difference may impact the ease of use of the sugar and/or the underlying defects in the pancreas^18^ and the propensity for low blood sugar in the *Smn^2B/−^* mice and patients.^9^ Overall, this study suggests that supplementation of nonfatty substrates for energy use is a key beneficial determinant of survival. Our findings strongly suggest that clinical nutritional guidelines need to be established from evidence‐based research to provide better care for SMA patients.

## Author Contribution

MOD designed study, performed experiments, main contributor for Figure 2. Analyzed data and created all figures. Wrote the manuscript. AT performed and provided support for experiments in Figure 2. LC provided support for experiments in Figure 2. AB provided support for experiments. RK designed study and prepared manuscript.

## Conflict of Interest

Marc‐Olivier Deguise received honoraria and travel accommodations by Biogen for the SMA Summit 2018 held in Montreal and the SMA academy 2019 held in Toronto, Canada. RK and the Ottawa Hospital Research Institute have a licensing agreement with Biogen for the *Smn^2B/−^* mouse model. These COI are outside the scope of this study. All other authors have no competing interests to declare.
